# Therapeutic delivery systems need to focus on cellular precision to target neurodegeneration

**DOI:** 10.1371/journal.pbio.3004049

**Published:** 2026-10-26

**Authors:** Lavanya Gupta, Sinem Agilkaya, Kim Susann Keller, Ebru Ercan-Herbst

**Affiliations:** School of Life Sciences, Reutlingen University, Reutlingen, Germany; School of Medicine and Health, Technical University, Munich, GERMANY

## Abstract

Crossing the blood–brain barrier is no longer a problem for delivering therapeutics to the brain. This Perspective argues that the main challenge is now how to deliver therapeutics to the right cells, in the right regions to target neurodegeneration, within a dynamically changing neurovascular unit.

## Teaser

The central problem when delivering drugs to the brain is no longer crossing the blood–brain barrier, but delivering therapeutics to the right cells, in the right regions to target neurodegeneration, within a dynamically changing neurovascular unit.

The blood–brain barrier (BBB) is a dynamic neurovascular interface; tightly sealed, polarized endothelial cells, pericytes, basement membrane, astrocytic end‑feet, transporters, and efflux systems working together to regulate the chemical composition of the central nervous system (CNS) and to restrict most therapeutics from entering brain tissue [1]. Strategies to overcome this barrier include a range of non-invasive approaches, such as exploiting endogenous transport pathways or transiently modulating the BBB [1–4], as well as invasive approaches that bypass the barrier altogether by delivering therapeutics directly into the brain parenchyma or cerebrospinal fluid [1]. Crossing the BBB is therefore no longer the principal obstacle to CNS drug delivery; instead, the challenge lies in achieving therapeutic efficacy after the barrier has been crossed. To meet this challenge, we need to shift our focus away from BBB penetration and toward cellular precision.

CNS drug delivery needs to be understood as a multi-step neurovascular delivery cascade (Fig 1), in which BBB recognition and crossing constitute only the initial stages. Before a therapeutic can engage its intracellular target, additional steps are required, including distribution within the brain parenchyma, uptake by the intended cell type, and escape from the endosome into the cytosol. These subsequent steps, rather than transport across the BBB, are the principal determinants of therapeutic outcome. As such, CNS drug delivery is increasingly becoming an engineering problem, rather than a binary question of whether a therapeutic can cross the BBB or not. To move forward, the field needs to stop asking whether a therapeutic reaches the brain and instead rethink what should constitute successful CNS drug delivery.

**Fig 1:**
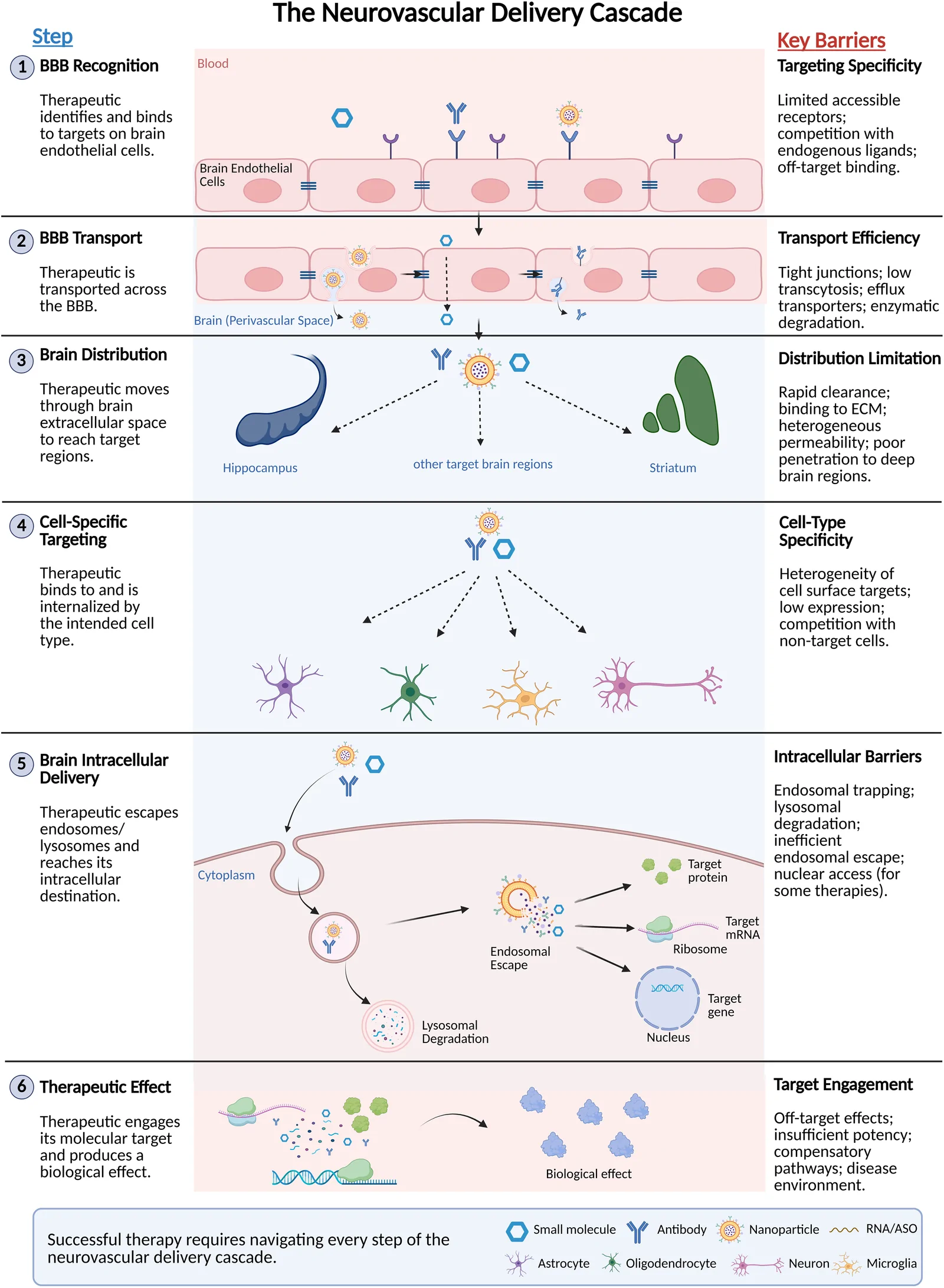
The neurovascular delivery cascade. Therapeutic delivery to a specific intracellular target within the central nervous system (CNS) requires successful completion of multiple sequential steps: (1) blood–brain barrier (BBB) recognition; (2) transport across the BBB; (3) brain distribution; (4) cellular targeting; (5) intracellular trafficking; and (6) target engagement. Failure at any stage of this cascade—even after successful passage across the BBB—depicted here as key barriers, can result in limited regional distribution, off-target cellular uptake, endosomal entrapment, lysosomal degradation, or insufficient target engagement, ultimately reducing therapeutic efficacy. Created in BioRender. Ercan Herbst, E. (2026) https://BioRender.com/r7uwgnf.

We would suggest that two current practices in the field need to be reconsidered. First, broad, untargeted biodistribution throughout the brain should not be interpreted as an indicator of successful delivery. A therapeutic that reaches most of the brain without cell-type selectivity increases the probability of off-target engagement and toxicity and is unlikely to improve outcomes in diseases defined by discrete, vulnerable cell populations. Thus, although systemic administration offers broad and minimally invasive access to the CNS, it may be suboptimal when pathological changes are highly restricted to specific brain regions or cell populations. In such cases, local administration may provide a more direct route to the affected region and enable higher local target exposure while potentially reducing systemic exposure. This is particularly relevant for neurodegenerative diseases, which do not affect the brain uniformly: Alzheimer’s disease predominantly involves the hippocampus and entorhinal cortex; Parkinson’s disease targets dopaminergic neurons of the nigrostriatal pathway; amyotrophic lateral sclerosis (ALS; also known as motor neuron disease) affects upper and lower motor neurons; and Huntington’s disease is characterized by striatal degeneration [5]. Even within these affected regions, target cell populations are heterogeneous, encompassing neurons, microglia, astrocytes, and oligodendrocytes. Moreover, single-cell atlas studies have shown that the neurovascular unit itself varies considerably in receptor expression across capillary, arterial, and venous endothelial cells, across brain regions, and between healthy and diseased states [6]. To optimize region and cell-target specificity, the growing availability of single-nucleus transcriptomic datasets from post-mortem human brain regions could be leveraged to identify therapeutic targets with greater precision. However, identifying the most disease-associated target does not necessarily identify the most therapeutically tractable one. Target selection should therefore also consider whether the protein can be effectively and safely modulated, or whether targeting another, more druggable component of the same pathway could achieve a similar therapeutic effect. Conventional approaches such as small molecules and antibodies may be suitable for accessible targets, whereas intracellular or poorly druggable targets may require approaches such as RNA-based therapeutics or targeted protein degradation.

Reaching the appropriate brain region and cell type is only the first step; therapeutics must then efficiently find their target cells. Generating cell-type-specific delivery will therefore require capsid- and receptor-engineering approaches capable of producing predictable, defined tropism rather than broad distribution. Adeno-associated virus capsid engineering can achieve durable, anatomically targeted transgene expression in patients with Parkinson’s disease [7], indicating that this level of precision is achievable through vector design. Consistent with this, receptor-binding affinity itself determines transport efficiency: reduced affinity for the transferrin receptor favors productive transcytosis, whereas high-affinity binding favors lysosomal degradation [2], indicating that receptor engineering should be prioritized over receptor engagement alone.

A critical and often underappreciated barrier lies inside the cell itself. Therapeutics must reach specific intracellular compartments, such as the cytosol, nucleus or ribosome, to have their effects. Both antisense oligonucleotides (ASOs) and mRNA delivered via lipid nanoparticles (LNP) face a major bottleneck: most endocytosed cargo is directed toward lysosomal degradation. LNP formulations achieve endosomal escape at a very low efficiency, with only about 1%–2% of internalized siRNA reaching the cytosol, making this the most significant unresolved barrier to nucleic-acid-based CNS therapeutics [8]. Despite these challenges, ASOs are able to reach the motor, striatal, and cortical neurons and reduce the amount of disease-associated proteins in ALS, Huntington’s disease, and Alzheimer’s disease, respectively [9], suggesting that the intracellular barrier is a major challenge, but not an absolute limitation. Closing this gap will require directly optimizing the lipid composition of the nanoparticle itself, together with the use of endosomolytic excipients and helper lipids that destabilize the endosomal membrane and release cargo into the cytosol before it is routed to lysosomal degradation. LNP formulations should be screened against functional endpoints, not uptake alone—using approaches like high-throughput library screening and single-cell RNA-sequencing to identify which formulations drive functional cargo expression at the individual-cell level.

The second practice in the field that needs to be reconsidered is related to how drug delivery approaches are tested. Many brain-targeted drug delivery systems are developed and optimized using models with a healthy BBB, even though their intended application is often the treatment of a diseased and aging brain. The critical question is therefore whether approaches developed in these models will perform similarly in patients. Even in the absence of overt disease, aging remodels the BBB through endothelial dysfunction, altered tight junction organization, and changes in transporter expression and function [10]. Furthermore, neurodegenerative diseases are associated with BBB dysfunction and neurovascular remodeling: in Alzheimer’s disease, this includes impaired cerebrovascular integrity and endothelial dysfunction; in Parkinson’s disease, aberrant angiogenesis and reduced tight junction and adherens junction protein expression; and in multiple sclerosis, neuroinflammation and vascular dysfunction [11]. These structural and functional alterations could influence therapeutic transport across the BBB, raising concerns that delivery strategies optimized in young, healthy model systems may not accurately predict performance in older patients with disease. The abundance, localization, and function of the targets could differ substantially between healthy and diseased barriers, potentially altering uptake, transcytosis, biodistribution, and therapeutic efficacy. A delivery strategy validated at the time of diagnosis may therefore perform differently in a patient as vascular remodeling and cell loss progress.

Neurodegenerative diseases thus present a challenge that generic delivery platforms do not adequately address: delivering therapeutics in a dynamic cellular and vascular environment that changes with disease progression. To address this challenge, delivery platforms for neurodegenerative diseases need to be validated across multiple disease stages, rather than at diagnosis alone. Furthermore, platform design needs to be matched to the anatomically and cellularly distinct features of each disease. Disease modeling platforms such as patient-derived induced pluripotent stem cell (iPSC) models, together with human single-cell omics data that recapitulate BBB and target-cell specific dysfunction and heterogeneity, should be incorporated earlier in preclinical evaluation as a disease-relevant testing platform for CNS delivery. By combining iPSC-derived BBB with specific target-cell populations, a controlled human-based model system for comparing therapeutic transport to specific cell type, barrier integrity, and permeability can be achieved.

In practice, CNS drug delivery should be viewed as a dynamic neurovascular delivery cascade, rather than a single BBB-crossing event. Research in recent years has already demonstrated that each level of this cascade—receptor engagement, regional targeting, cellular selectivity, and intracellular delivery—can be individually engineered and optimized. The next frontier lies not in solving these challenges in isolation, but in integrating them into unified delivery platforms capable of matching the right therapeutic to the right cell, in the right brain region, at the right time.

## Abbreviations

ALS: Amyotrophic lateral sclerosis
ASOs: antisense oligonucleotides
BBB: blood–brain barrier
CNS: central nervous system
iPSC: induced pluripotent stem cell
LNP: lipid nanoparticles
